# Supplementary material for: Improved estimates on global carbon stock and carbon pools in tidal wetlands
Source: Nat Commun. 2020 Jan 16;11:317. doi: 10.1038/s41467-019-14120-2 (PMC6965625; doi:10.1038/s41467-019-14120-2)
Supplement: Supplementary file 1 — Supplementary Information for “Improved estimates on global carbon stock and carbon pools in tidal wetlands” by Ouyang and Lee [file 41467_2019_14120_MOESM1_ESM.pdf]

Supplementary Information for "Improved estimates on global carbon stock and carbon pools in tidal wetlands" by Ouyang and Lee.

Supplementary Notes

Supplementary Figures

Supplementary Tables

Supplementary References

Supplementary Notes

We corrected the individual studies or reviews on sediment OC stocks using LOI as an estimate for OC with the conversion factor 1.724 (LOI:OC) or 0.58 (OC: organic matter). The relationship is polynomial for all coupled data on OC and LOI in mangroves due to the smaller slope for low LOI sediments and higher slope for high LOI sediments. Therefore, we further developed relationship for low (eq. 1) and high (eq. 2) LOI sediments in mangroves, respectively as below. We only present the two equations for here but the polynomial relationship is suitable for all sediments regardless of LOI levels.

$$OC = 0.26LOI + 0.01 \quad (1)$$

$$OC = 0.46LOI - 2 \quad (2)$$

Sediment IC contents of our field measurements were estimated from the constant dry weight of sediments, and sediment weights after combusted at 550 °C and 950 °C. When the sediments after LOI analysis were heated at 950 °C, carbonate transferred to CO_2, which determines the loss in sediment weight between 550 °C and 950 °C. The relationships between the different components are listed as below in eq. 3- eq. 6.

$$LOI= (W_{550}-W_{60})/W_{60}\times 100\% \quad (3)$$

$$LOI_{950}= (W_{550}-W_{950})/W_{60}\times 100\% \quad (4)$$

$$W_{carbonate} = 1.36\times LOI_{950} \quad (5)$$

$$W_{IC}=0.2\times W_{carbonate} \quad (6)$$

Where W_{60} denotes sediment dried at 60°C until constant weights. W_{550} and W_{950} denotes sediment weights after combusted at 550 °C and 950 °C, respectively. $W_{carbonate}$ denotes the weight percentage of carbonate in sediments. The coefficient 1.36 is the molecular weight ratio between carbonate (60) and CO_2 (44). W_{IC} denotes the weight percentage of sediment IC content. The coefficient 0.2 is the ratio of carbon atom in carbonate.

a)

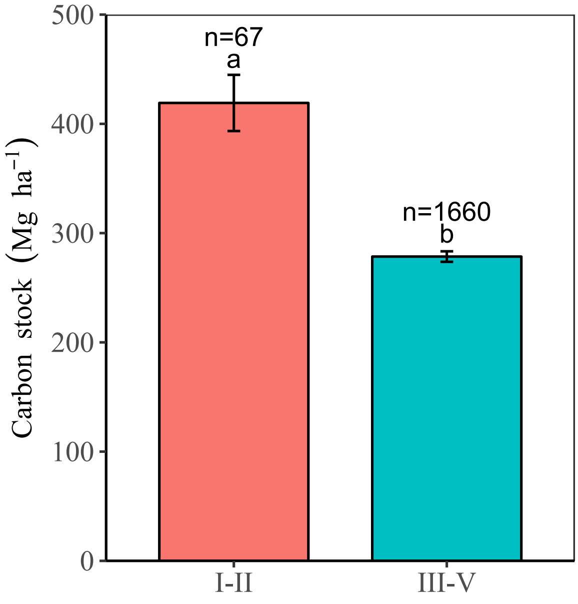

500
400
300
200
100
0
Carbon stock (Mg ha-1)
n=67
a
n=1660
b
I-II
III-V

b)

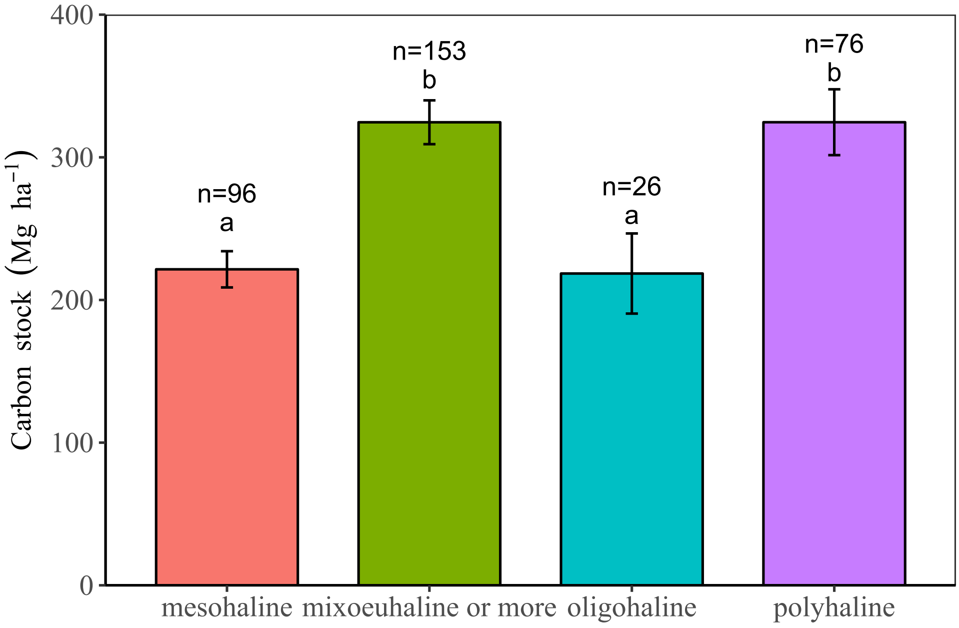

400
300
200
100
0
Carbon stock (Mg ha-1)
n=96
a
n=153
b
n=26
a
n=76
b
mesohaline
mixoeuhaline or more
oligohaline
polyhaline

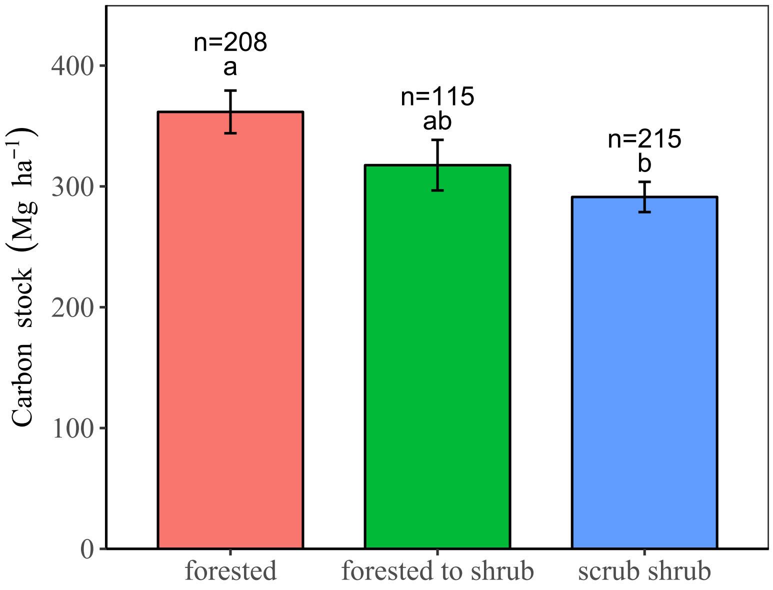

d)

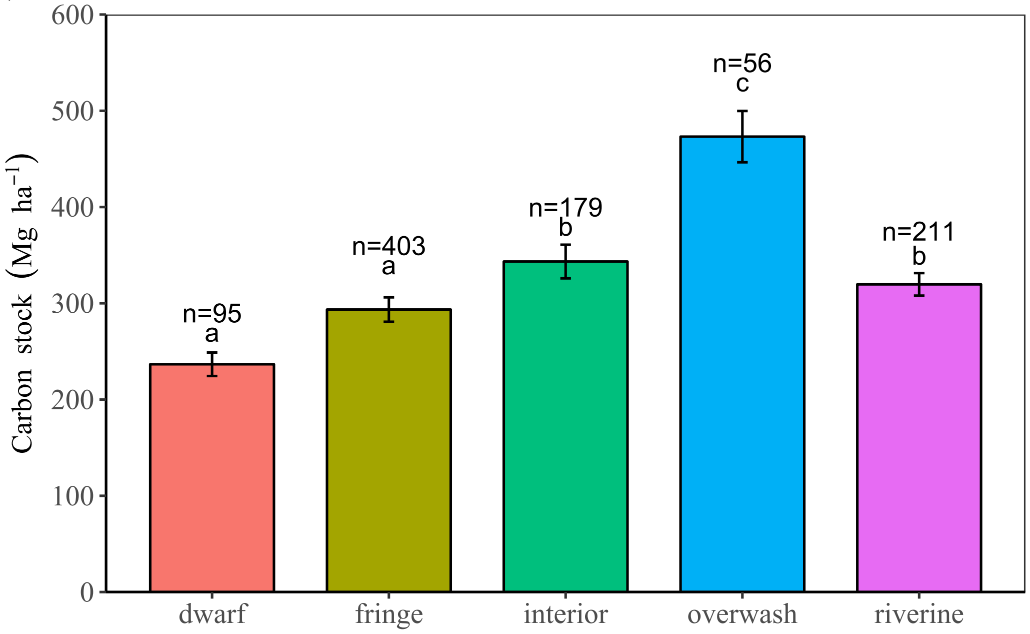

Supplementary Figure 1 Variation of sediment OC stocks with Holocene relative sea level rise zones (a), salinity categories (b), forest conditions (c) and mangrove types (d). Error bars represent mean ± standard error.

Supplementary Table 1 Literature data and field measurements of both OC and LOI in tidal wetlands

Sampling site	Wetland type	OC (%)	LOI (%)	References
Pichavaram mangrove, India	mangrove	0.06-1.97	0.21-7.53	Ranjan, et al. [1]
The coast of Singapore	mangrove	0.04-34.62	0.25-79.9	Bird, et al. [2]
Rubicon Estuary, Australia	saltmarsh	0.17-1.7	0.65-3.41	Beasy and Ellison [3]

San Francisco Bay, USA	saltmarsh	3.15-27.12	7.6-59.5	Callaway, et al. [4]
Snohomish estuary, USA	saltmarsh	0.51-39.89	2.26-74.12	Crooks, et al. [5]
Eastern North Carolina, USA	saltmarsh	0.11-27.5	0.2-50.9	Craft, et al. [6]
Esmeraldas, Ecuador	mangrove	0.6-27.2	3.4-39.5	DelVecchia, et al. [7]
Mui Ca Mau National Park, Vietnam	mangrove	1.2-5	2.8-17.1	Tue, et al. [8]
South Carolina, USA	saltmarsh	0.5-3.7	1.8-14	Morris and Whiting [9]
Louisiana, USA	saltmarsh	3.17-13.49	12.76-39.87	Piazza, et al. [10]
Florida Coastal Everglades, USA	mangrove	21.7-31.12	55.2-68.9	Castañeda-Moya, et al. [11]
Moreton Bay, Australia	mangrove	1.9	5.1	Hutchins, et al. [12]
Shark River, Florida, USA	mangrove	21.7	50.35	Chambers [13]
Hatiya Island, Bangladesh	mangrove	1.79-2.38	3.01-4.1	Shaifullah, et al. [14]
Gulf of Mexico, USA	mangrove	18-27	40-59	Breithaupt, et al. [15]
Auckland, New Zealand	mangrove	0.74-7.42	6.3-25.1	Woodroffe [16]
The Indus Delta, Pakistan	mangrove	0.48-1.15	2.9-5.4	Kristensen, et al. [17]
Hong Kong, China	saltmarsh<br>mangrove	0.25-1.45<br>0.04-25.41	2.53-6.65<br>0.95-56.51	This study
Ambanja and Ambaro, Madagascar	mangrove	0.36-8.39	2.38-16.15	Blue Ventures Unpublished in Jones, et al. [18]
Everglades, USA	mangrove	33.46	66.54	Chen and Twilley [19]
Agua Brava, Mexico	mangrove	1.45-2.79	2.79-4.83	Gutierrez, et al. [20]
Palau, Indonesia	mangrove	6.02-27.66	17.13-50.1	Kauffman, et al. [21]
Gazi Bay, Kenya	mangrove	0.81-9.59	2.4-21.08	Kazungu [22]
United Arab Emirates	mangrove<br>saltmarsh	0.19-29.78<br>0.21-9.18	2.27-62.9<br>2.67-19.8	Schile, et al. [23]
Sofala, Mozambique	mangrove	1.42-1.57	2.35-2.73	Sitoe, et al. [24]
Florida, Louisiana, Texas, USA	mangrove<br>saltmarsh	0.19-12.75<br>2.86-10.55	2.11-38.01<br>13.68-34.93	Yando, et al. [25]
Matang, Malaysia	mangrove	5.2-24.27	20.05-52.69	Adame, et al. [26]
Singapore	mangrove	0.36-7.97	1.87-27.8	Phang, et al. [27]
Campeche, Mexico	mangrove	0.3-7.15	0.5-13.9	Ramírez-Elías, et al. [28]
Mid-Atlantic USA	saltmarsh	6.3-30.2	14.1-60.8	Unger, et al. [29]

Connecticut, New York, USA	saltmarsh	7.94-23.73	18.33-49.5	Hill, et al. [30]

Supplementary Table 2 Literature data and field measurements of sediment IC stock in mangroves

Country	mean $CaCO_3$ concentration (mg cm^{-3})	Carbonate IC (mg cm^{-3})	IC stock (Mg ha^{-1})	References
Mexico	13.4-457.8	1.6-54.9	16.1-549.4	Adame, et al. [31]
Australia	19.7-224.2	2.4-26.9	23.6-269	Alongi, et al. [32]
Australia	5.2-736.6	0.6-88.4	6.3-883.9	Alongi, et al. [33]
Australia	14.8-1218.4	1.8-146.2	17.7-1462.1	Brunskill, et al. [34]
Australia	0-6.2	0-0.7	0-7.4	Brunskill, et al. [35]
Fed. States of Micronesia	0.3-11.7	0.04-1.4	0.4-14.1	Fujimoto, et al. [36]
Vietnam	9.2	1.1	11	Grellier, et al. [37]
China	1.8-77.8	0.2-9.3	2.2-93.4	Guan [38]
Kenya	7.8	0.9	9.3	Hemminga, et al. [39]
Japan	19.9	2.4	23.8	Higashi and Shinagawa [40]
Australia	12.2-28.6	1.5-3.4	14.7-34.3	Howe, et al. [41]
New Caledonia	0	0	0	Jacotot [42]
India	176.1	21.1	211.3	Kathiresan, et al. [43]
Bahamas	563.5	67.6	676.2	Koch and Madden [44]
French Guiana/Guadeloupe	119.8	14.4	143.8	Lallier-Verges, et al. [45]
Tanzania	0.7-3.4	0.08-0.4	0.8-4.1	Machiwa [46]
Egypt	446.3-841.2	53.6-100.9	535.5-1009.4	Madkour, et al. [47]
Kenya	0-4.1	0-0.5	0-4.9	Middelburg, et al. [48]
Egypt	331-332.4	39.7-39.9	397.2-398.8	Okbah, et al. [49]
Mexico	263.2	31.6	315.8	Ramírez-Elías, et al. [28]
United Arab Emirates	79-1255.7	9.5-150.7	94.8-1506.9	Schile, et al. [23]
Nigeria	85.9	10.3	103	Ukpong [50,]Ukpong [51]
USA	75-123.8	9-14.9	90-148.5	Yando, et al. [25]
Saudi Arabia	856-878	102.7-105.4	1027.5-1053.5	Saderne, et al. [52]
USA	77	9.3	92.8	
Australia	2-14	0.2-1.7	2.5-16.9	
Hong Kong, China	16-24	1.9-2.9	19.2-28.8	This study

Supplementary Table 3 Comparison of relationships between OC and LOI for coastal wetlands developed in this study with references

Type of coastal wetlands	Relationship between OC and LOI	Remarks	References
Estuarine marsh	$OC=(0.4\pm0.01)LOI+(0.025\pm0.0003)LOI^2$	Samples from North Carolina, USA	Craft, et al. [6]
Tidal wetlands	$OC=(0.0421\pm0.012)LOI+(0.074\pm0.014) LOI^2-0.0080\pm0.0021$	Samples from USA freshwater and saline tidal wetlands	Holmquist, et al. [53]
Mangroves	$OC=0.47LOI$	$OC=0.5LOI$ [54] was used to convert OC from LOI in their analysis. 146 samples from their global synthesis	Sanderman, et al. [55]
Mangroves	$OC=0.415LOI+2.89$	Used in Atwood et al. (2017) to convert OC from LOI in their analysis, samples from Palau, Indonesia	Kauffman, et al. [21]
Tidal salt marshes	$OC=0.47\%LOI+0.0008 LOI^2$	Samples from Maine, USA	Johnson et al. unpublished data
Mangroves	$OC=(0.21\pm0.01)LOI^{1.12\pm0.02}$	1189 samples from our global synthesis and field data	This study
Saltmarshes	$OC=0.52\pm0.006LOI-1.17\pm0.12$	Global, 344 samples from our global synthesis and field data	

Supplementary References

1 Ranjan, R. K. *et al.* Bulk organic matter characteristics in the Pichavaram mangrove – estuarine complex, south-eastern India. *Appl. Geochem.* **25**, 1176-1186, doi:10.1016/j.apgeochem.2010.05.003 (2010).

2 Bird, M. *et al.* Calculating sediment compaction for radiocarbon dating of intertidal sediments. *Radiocarbon* **46**, 421-435 (2004).

3 Beasy, K. M. & Ellison, J. C. J. I. J. o. B. Comparison of three methods for the quantification of sediment organic carbon in salt marshes of the Rubicon Estuary, Tasmania, Australia. *International Journal of Biology* **5**, 1-13 (2013).

4 Callaway, J. C. *et al.* Carbon sequestration and sediment accretion in San Francisco Bay tidal wetlands. *Estuar. Coast.* **35**, 1163-1181 (2012).

5 Crooks, S. *et al.* Coastal blue carbon opportunity assessment for the Snohomish Estuary: The climate benefits of estuary restoration. Report by Environmental Science Associates, Western Washington University, EarthCorps,. (2014).

6 Craft, C. *et al.* Loss on ignition and Kjeldahl digestion for estimating organic carbon and total nitrogen in estuarine marsh soils: calibration with dry combustion. *Estuaries* **14**, 175-179 (1991).

7 DelVecchia, A. G. *et al.* Organic carbon inventories in natural and restored Ecuadorian mangrove forests. *Peer J* **2**, e388, doi:10.7717/peerj.388 (2014).

8 Tue, N. T. *et al.* Carbon storage of a tropical mangrove forest in Mui Ca Mau National Park, Vietnam. *Catena* **121**, 119-126 (2014).

9 Morris, J. T. & Whiting, G. J. Emission of gaseous carbon dioxide from salt-marsh sediments and its relation to other carbon losses. *Estuaries* **9**, 9-19 (1986).

10 Piazza, S. C. *et al.* Geomorphic and ecological effects of Hurricanes Katrina and Rita on coastal Louisiana marsh communities. Report No. 2331-1258, (US Geological Survey, 2011).

11 Castañeda-Moya, E. *et al.* Allocation of biomass and net primary productivity of mangrove forests along environmental gradients in the Florida Coastal Everglades, USA. *For. Ecol. Manag.* **307**, 226-241, doi:10.1016/j.foreco.2013.07.011 (2013).

12 Hutchins, C. M. *et al.* The effect of manipulating sediment pH on the porewater chemistry of copper-and zinc-spiked sediments. **69**, 1089-1099 (2007).

13 Chambers, L. G., Davis, S. E., Troxler, T., Boyer, J. N., Downey-Wall, A., Scinto, L. J. Biogeochemical effects of simulated sea level rise on carbon loss in an Everglades mangrove peat soil. *Hydrobiologia* **726**, 195-211, doi:10.1007/s10750-013-1764-6 (2014).

14 Shaifullah, K. *et al.* Coastal afforestation effects on soil properties at Hatiya in Bangladesh. **20**, 243-248 (2009).

15 Breithaupt, J. L. *et al.* Temporal variability of carbon and nutrient burial, sediment accretion, and mass accumulation over the past century in a carbonate platform mangrove forest of the Florida Everglades. *J. Geophys. Res. Biogeosci.* **119**, 2032-2048, doi:10.1002/2014jg002715 (2014).

16 Woodroffe, C. D. Studies of a mangrove basin, Tuff Crater, New Zealand: I. Mangrove biomass and production of detritus. *Estuar. Coast. Shelf Sci.* **20**, 265-280, doi:10.1016/0272-7714(85)90042-3 (1985).

17 Kristensen, E. *et al.* Preliminary study of benthic metabolism and sulfate reduction in a mangrove swamp of the Indus Delta, Pakistan. *Mar. Ecol. Prog. Ser.* **90**, 287-297 (1992).

18 Jones, T. G. *et al.* in *Estuaries: A Lifeline of Ecosystem Services in the Western Indian Ocean* 67-85 (Springer, 2016).

19 Chen, R. & Twilley, R. R. J. B. A simulation model of organic matter and nutrient accumulation in mangrove wetland soils. *Biogeochemistry* **44**, 93-118 (1999).

20 Gutierrez, J. *et al.* Comparison of the mangrove soil with different levels of disturbance in tropical Agua Brava Lagoon, Mexican pacific. **14**, 45-57 (2016).

21 Kauffman, J. B. *et al.* Ecosystem carbon stocks of Micronesian mangrove forests. *Wetlands* **31**, 343-352, doi:10.1007/s13157-011-0148-9 (2011).

22 Kazungu, J. M. *Nitrogen-transformational processes in a tropical mangrove ecosystem (Gazi Bay, Kenya). PhD Thesis. VUB: Belgium*, (1996).

23 Schile, L. M. *et al.* Limits on carbon sequestration in arid blue carbon ecosystems. **27**, 859-874 (2017).

24 Sitoe, A. *et al.* Biomass and carbon stocks of Sofala bay mangrove forests. *Forests* **5**, 1967-1981 (2014).

25 Yando, E. S. *et al.* Salt marsh-mangrove ecotones: using structural gradients to investigate the effects of woody plant encroachment on plant–soil interactions and ecosystem carbon pools. *J. Ecol.* **104**, 1020-1031, doi:10.1111/1365-2745.12571 (2016).

26 Adame, M. F. *et al.* Loss and recovery of carbon and nitrogen after mangrove clearing. *Ocean Coast. Manag.* **161**, 117-126, doi:10.1016/j.ocecoaman.2018.04.019 (2018).

27 Phang, V. X. H. *et al.* Ecosystem carbon stocks across a tropical intertidal habitat mosaic of mangrove forest, seagrass meadow, mudflat and sandbar. *Earth Surf. Process. Landforms* **40**, 1387-1400, doi:10.1002/esp.3745 (2015).

28 Ramírez-Elías, M. A. *et al.* Identification of culturable microbial functional groups isolated from the rhizosphere of four species of mangroves and their biotechnological potential. *Appl. Soil Ecol.* **82**, 1-10, doi:10.1016/j.apsoil.2014.05.001 (2014).

29 Unger, V. *et al.* Stability of organic carbon accumulating in Spartina alterniflora-dominated salt marshes of the Mid-Atlantic US. **182**, 179-189 (2016).

30 Hill, T. D. *et al.* Coastal wetland response to sea level rise in Connecticut and New York. **163**, 185-193 (2015).

31 Adame, M. F. *et al.* Carbon stocks of tropical coastal wetlands within the karstic landscape of the Mexican Caribbean. *PLoS One* **8**, e56569 (2013).

32 Alongi, D. *et al.* Below-ground decomposition of organic matter in forests of the mangroves *Rhizophora stylosa* and *Avicennia marina* along the arid coast of Western Australia. *Aquat. Bot.* **68**, 97-122 (2000).

33 Alongi, D. M. *et al.* Growth and development of mangrove forests overlying smothered coral reefs, Sulawesi and Sumatra, Indonesia. *Mar. Ecol. Prog. Ser.* **370**, 97-109 (2008).

34 Brunskill, G. J. *et al.* Geochemistry and particle size of surface sediments of Exmouth Gulf, Northwest Shelf, Australia. *Cont. Shelf Res.* **21**, 157-201, doi:10.1016/S0278-4343(00)00076-5 (2001).

35 Brunskill, G. J. *et al.* Carbon burial rates in sediments and a carbon mass balance for the Herbert River region of the Great Barrier Reef continental shelf, North Queensland, Australia. *Estuar. Coast. Shelf S.* **54**, 677-700 (2002).

36 Fujimoto, K. *et al.* Belowground carbon storage of Micronesian mangrove forests. *Ecol. Res.* **14**, 409-413, doi:10.1046/j.1440-1703.1999.00313.x (1999).

37 Grellier, S. *et al.* Changes in soil characteristics and C dynamics after mangrove clearing (Vietnam). *Sci. Total Environ.* **593-594**, 654-663, doi:10.1016/j.scitotenv.2017.03.204 (2017).

38 Guan, W., Xiong, Y., & Liao, B. . Soil inorganic carbon in mangroves of tropical China: patterns and implications. *Biology letters* **14**, 20180483 (2018).

39 Hemminga, M. A. *et al.* Carbon outwelling from a mangrove forest with adjacent seagrass beds and coral reefs (Gazi Bay, Kenya). *Mar. Ecol. Prog. Ser.* **106**, 291-301 (1994).

40 Higashi, T. & Shinagawa, A. Soils of a mangrove forest composed of *Rhizophora mucronata* and *Bruguiera gymnorrhiza* from Ishigaki Island, Japan. *Soil Sci. Plant Nutr.* **31**, 427-435, doi:10.1080/00380768.1985.10557450 (1985).

41 Howe, A. J. *et al.* Surface evolution and carbon sequestration in disturbed and undisturbed wetland soils of the Hunter estuary, southeast Australia. *Estuar. Coast. Shelf Sci.* **84**, 75-83, doi:10.1016/j.ecss.2009.06.006 (2009).

42 Jacotot, A., Marchand, C., Rosenheim, B. E., Domack, E. W., & Allenbach, M. Mangrove sediment carbon stocks along an elevation gradient: Influence of the late Holocene marine regression (New Caledonia). *Mar. Geol.* **404**, 60-70 (2018).

43 Kathiresan, K. *et al.* Impact of mangrove vegetation on seasonal carbon burial and other sediment characteristics in the Vellar-Coleroon estuary, India. *J. For. Res.* **25**, 787-794, doi:10.1007/s11676-014-0526-2 (2014).

44 Koch, M. S. & Madden, C. J. Patterns of primary production and nutrient availability in a Bahamas lagoon with fringing mangroves. *Mar. Ecol. Prog. Ser.* **219**, 109-119 (2001).

45 Lallier-Verges, E. *et al.* Relationships between environmental conditions and the diagenetic evolution of organic matter derived from higher plants in a modern

mangrove swamp system (Guadeloupe, French West Indies). *Org. Geochem.* **29**, 1663-1686, doi:10.1016/S0146-6380(98)00179-X (1998).

46 Machiwa, J. F. Distribution and remineralization of organic carbon in sediments of a mangroves stand partly contaminated with sewage waste. *Ambio* **27**, 740–744 (1998).

47 Madkour, H. A. *et al.* Environmental texture and geochemistry of the sediments of a subtropical mangrove ecosystem and surrounding areas, Red Sea Coast, Egypt. *Arab J. Geosci.* **7**, 3427-3440, doi:10.1007/s12517-013-0977-3 (2014).

48 Middelburg, J. J. *et al.* Sediment biogeochemistry in an East African mangrove forest (Gazi Bay, Kenya). *Biogeochemistry* **34**, 133-155, doi:10.1007/bf00000899 (1996).

49 Okbah, M. A. *et al.* Geochemical forms of trace metals in mangrove sediments—Red Sea (Egypt). *Chem. Ecol.* **21**, 23-36, doi:10.1080/02757540512331323953 (2005).

50 Ukpong, I. E. Ecological classification of Nigerian mangroves using soil nutrient gradient analysis. *Wetl. Ecol. Manag.* **8**, 263-272, doi:10.1023/a:1008452923256 (2000).

51 Ukpong, I. E. An ordination study of mangrove swamp communities in West Africa. *Vegetatio* **116**, 147-159, doi:10.1007/bf00045305 (1995).

52 Saderne, V. *et al.* Role of carbonate burial in Blue Carbon budgets. *Nat. Commun.* **10**, 1106, doi:10.1038/s41467-019-08842-6 (2019).

53 Holmquist, J. R. *et al.* Accuracy and precision of tidal wetland soil carbon mapping in the conterminous United States. *Sci. Rep.* **8**, 9478 (2018).

54 Pribyl, D. W. A critical review of the conventional SOC to SOM conversion factor. *Geoderma* **156**, 75-83, doi:10.1016/j.geoderma.2010.02.003 (2010).

55 Sanderman, J. *et al.* A global map of mangrove forest soil carbon at 30 m spatial resolution. *Environ. Res. Lett.* (2018).

56 Kristensen, E. *et al.* Organic carbon dynamics in mangrove ecosystems: a review. *Aquat. Bot.* **89**, 201-219 (2008).

57 Donato, D. C. *et al.* Mangroves among the most carbon-rich forests in the tropics. *Nat. Geosci.* **4**, 293-297, doi:10.1038/ngeo1123 (2011).

58 Chmura, G. L. *et al.* Global carbon sequestration in tidal, saline wetland soils. *Global Biogeochem. Cy.* **17**, 1111, doi:10.1029/2002GB001917 (2003).

59 Jardine, S. L. & Siikamäki, J. V. A global predictive model of carbon in mangrove soils. *Environ. Res. Lett.* **9**, 104013, doi:10.1088/1748-9326/9/10/104013 (2014).

60 Atwood, T. B. *et al.* Global patterns in mangrove soil carbon stocks and losses. *Nat. Clim. Chang.* **7**, 523-528 (2017).

61 Simard, M. *et al.* Mangrove canopy height globally related to precipitation, temperature and cyclone frequency. *Nat. Geosci.* **12**, 40-45, doi:10.1038/s41561-018-0279-1 (2019).

62 Tang, W. *et al.* Big Geospatial Data Analytics for Global Mangrove Biomass and Carbon Estimation. *Sustainability* **10**, 472 (2018).

63 Hamilton, S. E. & Friess, D. A. Global carbon stocks and potential emissions due to mangrove deforestation from 2000 to 2012. *Nat. Clim. Chang.* **8**, 240-244, doi:10.1038/s41558-018-0090-4 (2018).

64 Twilley, R. R. *et al.* Carbon sinks inmangroves and their implications to carbon budget of tropical coastal ecosystems. *Water Air Soil Pollut.* **64**, 265-288 (1992).

65 Hutchison, J. *et al.* Predicting global patterns in mangrove forest biomass. *Conserv. Lett.* **7**, 233-240 (2014).
