# Supplementary material for: Improved estimates on global carbon stock and carbon pools in tidal wetlands
Source: Nat Commun. 2020 Jan 16;11:317. doi: 10.1038/s41467-019-14120-2 (PMC6965625; doi:10.1038/s41467-019-14120-2)
Supplement: Supplementary file 3 — Description of additonal sup files [file 41467_2019_14120_MOESM3_ESM.docx]

Description of additional supplementary files

Title: Dataset 1

Description: a dataset on the sediment IC stocks of the global database

Title: Dataset 2

Description: a dataset on the sediment OC stocks at site levels of the global database

Title: Dataset 3

Description: a dataset on all observation on the sediment OC stocks with site, core and depth information

Title: Dataset 4

Description: a dataset on all data on coupled measurements of OC and LOI in our global database

Title: Dataset 5

Description: a dataset on downed/dead wood carbon stock in our global data base

Title: Dataset 6

Description: a dataset on dead root carbon stock in our global data base

Title: Dataset 7

Description: a dataset on litter carbon stock in our global data base

Title: Dataset 8

Description: a list of all references on dead biomass carbon (downed/dead wood, dead root and litter carbon stock)

Title: Dataset 9

Description: a list of all references on sediment OC stocks
